# Medical patch disposal practices and information sources: A pharmacy customer survey before and after an information initiative

**DOI:** 10.1016/j.rcsop.2026.100841

**Published:** 2026-09-12

**Authors:** Lasse Alajärvi, Outi Lapatto-Reiniluoto, Henna Kyllönen, Piia Siitonen

**Affiliations:** a School of Pharmacy, Faculty of Health Sciences, University of Eastern Finland, Kuopio, Finland; b Department of Clinical Pharmacology, University of Helsinki, Helsinki, Finland; c HUS Pharmacy, Helsinki University Hospital, Helsinki, Finland; d Clinical Pharmacy Group, Faculty of Pharmacy, University of Helsinki, Helsinki, Finland

**Keywords:** Disposal practices, Medical patch, Counselling, Patient education, Sustainable healthcare

## Abstract

**Background:**

Promoting proper disposal of pharmaceuticals is among the most essential means to mitigate their environmental impacts. Several challenges prevent the proper disposal of medicines, such as a lack of guidance and difficulties in implementing the guidance. These issues are pronounced in the case of medical patch disposal, which is yet an unexplored subject in research. This study examined pharmacy customers' practices, information sources, and the impact of a national half-year-long information campaign regarding the proper disposal of medical patches.

**Materials and methods:**

A cross-sectional survey was conducted among Finnish pharmacy customers purchasing medical patches before (spring 2022, *n* = 176) and after (spring 2023, *n* = 254) a nationwide information campaign launched in autumn 2022. Data were collected through structured questionnaires administered by pharmacy students during community pharmacy internships. Disposal practices, information sources, and customer preferences were analyzed using descriptive statistics and Chi-square tests.

**Results:**

Of the respondents (*N* = 430), 80.1% stated to dispose of the unused patches correctly by returning them to a pharmacy, while only 40.4% did this with used patches. Approximately half (52.9%) of the respondents recalled having received information about correct medical patch disposal, with 81.7% of these considering it sufficient. However, significant disparities existed across patch types: memory disorder patch users were less likely to have received information (29.8% vs. 60.6% for opioid patches, *p* < 0.001) or consider it adequate (43.8% vs. 87.9%, *p* < 0.001). Pharmacies were both the most common actual source (73.6%) and preferred source (91.3%) of disposal information. Younger respondents (<45 years) more frequently sought information from multiple sources including package leaflets (58.1%), physicians (69.8%), and educational institutions. A slight, non-significant improvement in pharmacy return rates was observed between 2022 and 2023.

**Conclusions:**

This study showed only a modest improvement in medical patch disposal practices due to the information campaign, indicating the need for longer-term communicational measures to raise public awareness and promote proper disposal practices. These measures should employ diverse information channels and target different stakeholders, including the education of healthcare professionals.

## Highlights

• Medical patches pose unique challenges for proper pharmaceutical disposal.
• 80.3% properly disposed of unused patches, while only 40.8% did so with used ones.
• Pharmacies and physicians were the preferred sources for disposal guidance.
• Half recalled receiving guidance, mainly from pharmacies and package leaflets.
• Continuous, multi-channel communication is needed for all stakeholders.

## 1 Introduction

The environmental impact of pharmaceuticals is a significant global concern.^1^ Environmental residues from pharmaceuticals pollute water bodies, cause biodiversity loss, and contribute to antimicrobial resistance, for instance. As the well-being of societies depends on a healthy environment, it is crucial to prevent environmental pollution from pharmaceuticals. The main contributor to this environmental burden is the normal use and disposal of medicines.^2^ Healthcare professionals play a key role in informing the public about the correct use and disposal of medicines, and environmental considerations have already been implemented in the training of healthcare professionals.^3^ In addition to providing information, physicians, nurses, home-care staff, and pharmacists engage in the prevention of pharmaceutical waste and their environmental impacts by promoting rational use of medicines and by committing to appropriate storage and disposal practices of medicines.^4^ Even though considerations of environmental aspects have become more common regarding healthcare and pharmaceuticals, the level of knowledge regarding the environmental impacts of pharmaceuticals and their causes is low among the public, and research on the topic is scarce.^5^

Correct disposal methods of pharmaceuticals are essential to prevent the compounds from ending up in the environment. Therefore, regulatory measures have been taken to control the environmental impact of pharmaceutical waste. For example, in the EU, Directive 2004/27/EC obliges countries to organise the separate collection of pharmaceutical waste for proper disposal. The implementation of this directive varies among EU member states, thereby limiting the detail of information that can be provided about the disposal of medicines in their package leaflets. The instructions on the package leaflet are general and can be, for instance, “Any unused medicinal product or waste material should be disposed of in accordance with local requirements”.^6^ Therefore, a medicine's product information usually lacks practical information on proper disposal. Many EU countries, including Finland, have implemented the collection of pharmaceutical waste through community pharmacies, which places a significant responsibility on the pharmacies to inform their customers about proper disposal practices.^7^

In the case of the environmental impact of pharmaceuticals, successful communication regarding the correct disposal of medicines has the potential to engage the public in more sustainable practices. This underlines the importance of pharmacies in raising awareness of this issue. In a healthcare context, information is considered to be associated closely with an individual's motivation to act.^8^ However, regarding individuals and the correct disposal of pharmaceutical waste, information, motivation, and behavioral skills may not be enough to bridge the knowledge–action gap resulting from, e.g., practical barriers, such as the perceived inconvenience of getting to the collection point or storing the medicines at home appropriately before disposal.^9^ Intention to correctly dispose of one's medicines may also depend on attitudes, perceived norms, behavioral control, and busyness regarding the issue.

Previous literature suggests that the medicine formulation may affect the disposal method chosen by its user.^10^, ^11^, 12. Some formulations may be perceived to pose a higher safety risk for health if not disposed of immediately, or the person may feel tempted to recycle the outer packaging of a liquid medicine, for instance, thereby pouring the medicine down the drain. Medical patches represent a formulation with particular safety and environmental considerations, as used patches retain substantial residual active pharmaceutical ingredients.^13^ Thereby, accidental exposure to, e.g., a highly potent opioid, fentanyl, in the form of a patch, can result in severe health outcomes. In addition to accidental exposures, the amounts of fentanyl in used patches, even after 3 days of use, are still sufficient for misuse purposes, highlighting the significance of their safe disposal.^14^ The remaining quantities of active pharmaceutical ingredients in medical patches after their intended use can also affect the environment if disposed of inappropriately. For example, estrogen and nicotine, commonly used pharmaceuticals in medical patches, are known to pose a risk for the aquatic environment.15., 16. Despite the obvious environmental risks, there is no previous research solely focused on disposal methods of medical patches, people's awareness of the correct disposal of them, nor the efficacy of communicational measures in improving disposal methods among their users.

### 1.1 Study aims and research questions

This study aimed to investigate pharmacy customers' disposal practices for medical patches in Finland, their knowledge and information needs regarding the issue, and to compare these results after a national pharmacy-based information campaign on the correct disposal of pharmaceuticals.

The specific research questions of this study are**:**

1. How do users of medical patches dispose of their patches?
2. Do users report sufficient knowledge of correct patch disposal methods?
3. Which previous and preferred information sources do users rely on for disposal guidance?
4. Did the results differ before and after a national pharmacy-based information campaign in promoting the correct disposal of pharmaceuticals?

## 2 Materials and methods

### 2.1 Study context

Finland has a community pharmacy system of 827 privately owned pharmacies with a ratio of approximately one pharmacy per 6722 inhabitants and at least one pharmacy in almost every municipality.^17^ Finnish pharmacies typically operate 58 h weekly, averaging 10 h daily.^18^ In Finland, the Medicines Act obliges pharmacies to provide patient counselling to promote their safe and proper use, including guidance on proper disposal of expired, used or unnecessary medicines. Pharmaceutical waste is classified as hazardous waste by the Government Decree on Waste 978/2021 appendix III, and households can return it to community pharmacies free of charge.^19^ According to a national survey conducted in Finland in 2019, approximately 90% of Finnish adults follow the correct disposal method of expired or unwanted pharmaceuticals.^10^

### 2.2 Data collection

The data was collected in two phases: in spring 2022, before an information campaign on proper disposal of medical patches in Finland and in spring 2023, after the campaign. The campaign provided information material for pharmacies to support patient counselling on the proper disposal of medical patches. Based on the annual pharmacy surveys, approximately 80% of Finnish pharmacies utilised these materials during autumn 2022 (personal communication, The Association of Finnish Pharmacies, Pharmaceutical Department, September 2024). All campaign materials remained available for pharmacies to use in their patient counselling. The data was collected as an obligatory assignment among third-year pharmacy students of the University of Eastern Finland completing their second community pharmacy internship period in spring 2022 and 2023. In total, 93 students participated it in 2022 and 127 in 2023. Each student was instructed to complete one to two assignments during their internship.

The study employed convenience sampling at the pharmacy level, whit participating pharmacies were determined by the locations where pharmacy students had independently arranged their internship placements. Participating pharmacies were located across all major regions of Finland, including both urban and rural areas. Some students completed their internship in the same pharmacy, but the majority were placed in different pharmacies. Therefore, the number of students closely corresponds to the number of the pharmacies. No formal sample size calculation was performed due to the exploratory nature of the study and the practical constraints of student-led data collection. The final sample size (*n* = 430) provides adequate statistical power for detecting medium effect sizes in the primary comparisons.

The students were instructed (Appendix A) to inform customers purchasing a prescription medical patch, either for personal use or on behalf of another user, about the study and invite them to participate. The data collection was conducted using a structured customer questionnaire (Appendix A) consisting of six structured multiple-choice questions. The questionnaire was designed to assess information on the customers' disposal practices of medical patches and their state of being informed about the issue, as well as information needs and information source preferences for it. Participants completed the questionnaire in paper format after the prescription dispensing situation. Due to the nature of the data collection, the response and refusal rates were not recorded.

### 2.3 Statistical analysis

Respondent's gender, age and the active pharmaceutical ingredient of the medical patch were used as background characteristics in the analysis of the results. Descriptive analysis was conducted using frequencies and crosstabulation. Questions allowing multiple responses (information sources) were analyzed using multiple response frequency tables, with percentages calculated as the proportion of respondents selecting each option. Differences between groups were analyzed using the Chi-square test and Fisher's exact test, with statistical significance set at *p*-value <0.05. All statistical analyses were performed using IBM SPSS Statistics version 29.0.2.0.

Medical patches were categorised into three groups based on their active pharmaceutical ingredients: opioids (buprenorphine or fentanyl), hormonal patches (estradiol, estradiol/norethisterone or norelgestromine/ethinyl estradiol), and patches used for memory disorder (rivastigmine or rotigotine). A fourth category, “Other patches” (containing lidocaine, lidocaine/prilocaine, scopolamine, glyceryl nitrate, or nicotine), was excluded from statistical analysis due to the low number of responses (*n* = 13).

Open-ended responses regarding disposal methods for medical patches were analyzed using inductive content analysis.^20^ Responses typically consisted of brief, unambiguous statements (e.g., single words or short phrases describing disposal methods). The unit of analysis was a word or a short sentence. Coding was performed by one researcher (PS), with the simple response structure minimising ambiguity in classification. “Burning” and “Healthcare unit is responsible for disposal” emerged as additional disposal options not included in the structured response alternatives.

“Burning” and “Healthcare unit is responsible for disposal” emerged as additional disposal options. For statistical analysis, disposal methods for used medical patches (in household waste, flushing down the drain, burning, and the healthcare unit being responsible for disposal) were combined into the category “Other method”. Similarly, disposal methods for unused and expired medical patches (in household waste, flushing down the drain, burning, or giving to another person to use) were also categorised as “Other method”. The frequencies in these individual categories were small, and they were combined into the “Other method” category to ensure adequate cell sizes for statistical testing (minimum expected frequency > 5). For descriptive statistics the questions about received information on medical patches were categorised into classes “Yes” and “No”. The option “I do not know” was excluded from the analysis.

### 2.4 Validity and reliability

The questionnaire was developed based on previous research on medicine disposal practices and information needs regarding the environmental impacts of pharmaceuticals.^5^, ^10^, ^27^ It was reviewed by pharmacy professionals and researchers familiar with survey studies (*n* = 5) to assess face and content validity, leading to minor revisions prior to implementation.

### 2.5 Ethical statement

The study settings and research process complied with national ethical instructions for non-medical human sciences research.^21^ Based on the national ethical guidelines, the study did not require a preliminary ethical review, as it was outside the scope of non-medical human sciences research for which such review is required in Finland. Participants were verbally informed about the research purpose and that taking part in this study was entirely voluntary, and they indicated their informed consent by participating in the survey.

## 3 Results

In total, 433 pharmacy customers participated in the study in 2022 and 2023 (Table 1). The final analysis included 430 respondents, after excluding three responses provided by care home nurses who answered from their unit's perspective. The respondents were between 20 and 93 years of age (mean 62.9, median 63, SD 14.5), and 72.7% of them were women. Of the dispensed medical patches (two years counted together), 57.0% were opioids (buprenorphine or fentanyl), 27.7% hormonal patches (estradiol, estradiol/norethisterone or norelgestromine/ethinyl estradiol), and 11.4% patches used for memory disorder (rivastigmine or rotigotine). In terms of geographical coverage of the pharmacies, all areas of mainland Finland were represented in this study.

**Table 1:** Characteristics of the participants (*N* = 430).

	2022 (*n* = 176)	2023 (*n* = 254)	Total (*N* = 430)
	% (*n*)	% (*n*)	% (*N*)
**Age (years)**			
−45	6.8 (12)	12.6 (32)	10.2 (44)
46–65	48.3 (85)	44.9 (114)	46.3 (199)
66–	44.9 (79)	42.5 (108)	43.5 (187)
**Gender**			
Female	73.3 (129)	72.4 (184)	72.8 (313)
Male	24.6 (44)	26.4 (67)	25.8 (111)
Not willing to respond	1.7 (3)	1.2 (3)	1.4 (6)
**Type of the medical patch**			
Opioid	56.3 (99)	57.5 (146)	57.0 (245)
Hormonal	26.7 (47)	29.9 (76)	28.6 (123)
Memory disorder	15.3 (27)	8.7 (22)	11.4 (49)
Other⁎	1.7 (3)	3.9 (10)	3.0 (13)

⁎ lidocaine, lidocaine/prilocaine, scopolamine, glyceryl nitrate, nicotine.

### 3.1 Disposal practices of medical patches

Of the respondents, 52.1% reported that they disposed of used medical patches in household waste or by other methods, while 40.4% returned them to the pharmacies (Table 2). For unused or expired patches, the majority of respondents (80.1%) returned them to pharmacies, while 17.5% disposed of them in household waste or by other methods. No statistically significant differences in disposal methods were observed between 2022 and 2023.

**Table 2:** Disposal of used and unused or expired medical patches in 2022 and 2023 among respondents with prior use of medical patches (*n* = 428).

	2022 (*n* = 174)	2023 (*n* = 254)	Total (*n* = 428)
**How do you usually dispose of used medical patches?**	**% (*n*)**	**% (*n*)**	**% (*n*)**
In household waste	54.0 (94)	50.8 (129)	52.1 (223)
Returning to pharmacy	37.9 (66)	42.1 (107)	40.4 (173)
Burning⁎	6.3 (11)	6.3 (16)	6.3 (27)
Flushing down the drain	0.0 (0)	0.4 (1)	0.2 (1)
The healthcare unit is responsible for disposal⁎	1.7 (3)	0.4 (1)	0.9 (4)
**How would you dispose of unused or expired medical patches?**			
Returning to pharmacy	76.0 (133)	83.0 (210)	80.1 (343)
In household waste	21.1 (37)	15.0 (38)	17.5 (75)
Flushing down the drain	0.0 (0)	0.4 (1)	0.2 (1)
Give to another person	0.6 (1)	0.0 (0)	0.2 (1)
Burning⁎	2.3 (4)	1.6 (4)	1.9 (8)

⁎ Emerged as additional disposal options from open-ended responses.

Respondents stated that they returned used opioid medical patches to a pharmacy more likely than hormonal patches or patches for memory disorder (50.8%, 29.3% and 22.4%, respectively, *p* < 0.001) (Table 3). Corresponding shares for disposal of unused or expired medical patches were 82.8% (opioid), 78.7% (hormonal) and 73.5% (memory disorder). There were no statistically significant differences between disposal methods in 2022 and 2023.

**Table 3:** Disposal practices of opioid, hormonal, and memory disorder patches in 2022 and 2023 among respondents with prior use of medical patches and responsible for their disposal by themselves (*n* = 412).

		Pharmacy % (*n*)	Other method⁎ % (*n*)	Pharmacy % (*n*)	Other method⁎ % (*n*)	Pharmacy % (*n*)	Other method⁎ % (*n*)
		2022 (*n* = 169)		2023 (*n* = 243)		Total (*n* = 412)	
**How do you usually dispose of used medical patches?**							
		*p* = 0.001		*p* = 0.014		*p* *<* 0.001	
	Opioid	51.6 (49)	48.4 (46)	50.3 (73)	49.7 (72)	50.8 (122)	49.2 (118)
	Hormonal	23.4 (11)	76.6 (36)	34.9 (25)	67.1 (51)	29.3 (36)	70.7 (87)
	Memory disorder	18.5 (5)	81.5 (22)	27.3 (6)	72.7 (16)	22.4 (11)	77.6 (38)
**Total**		**38.5 (65)**	**61.5 (104)**	**42.8 (104)**	**57.2 (139)**	**41.0 (169)**	**59.0 (243)**
		**2022 (*n*** **=** **172)**		**2023 (*n*** **=** **243)**		**Total (*n*** **=** **415)**	
**How would you dispose of** **unused or expired medical patches?**							
				*p* = 0.040			
	Opioid	74.5 (73)	25.5 (25)	88.4 (129)	11.6 (17)	82.8 (202)	17.2 (42)
	Hormonal	80.9 (38)	19.1 (9)	77.3 (58)	22.7 (17)	78.7 (96)	21.3 (26)
	Memory disorder	74.1 (20)	25.9 (7)	72.7 (16)	27.3 (6)	73.5 (36)	26.5 (13)
**Total**		**76.2 (131)**	**23.8 (41)**	**83.5 (203)**	**16.5 (40)**	**80.5 (334)**	**19.5 (81)**

⁎ The options for the question “How do you usually dispose of used medical patches?”: In household waste, Flushing down the drain, Burning, or Healthcare unit is responsible for disposal. The options for the question “How would you dispose of unused or expired medical patches?”: In household waste, Flushing down the drain, Burning, or Giving to another person to use.

### 3.2 Received information

Of the respondents, 52.6% in 2022, and 53.1% in 2023 reported that they had received information about disposal of medical patches (Table 4). Compared to other respondents, those using patches for a memory disorder were less likely to report having received information about the disposal of medical patches (29.8% vs 60.6%, 48.8%, *p* < 0.001) in total. No statistically significant differences exist in received information based on other background variables or between the years 2022 and 2023.

**Table 4:** Respondents' perceptions about received information on disposal of medical patches in 2022 and 2023.

		2022	2023	Total
		Yes % (*n*)	Yes % (*n*)	Yes % (*n*)
**Have you been previously informed about the disposal of medical patches?**		***n*** **=** **175**	***n*** **=** **243**	***n*** **=** **418**
		**52.6 (92)**	**53.1 (129)**	**52.9 (221)**
**Gender**				
	Female	50.8 (65)	51.7 (90)	51.3 (155)
	Male	54.5 (22)	56.1 (37)	55.5 (61)
**Age (years)**				
	−45	75.0 (9)	53.3 (16)	59.5 (25)
	46–65	52.9 (45)	56.6 (64)	55.1 (109)
	66–	48.7 (38)	49.0 (49)	48.9 (87)
	**Type of the medical patch**	*p* *=* 0.011	*p* *=* 0.005	*p* < 0.001
	Opioid	63.3 (62)	58.7 (81)	60.6 (143)
	Hormonal	42.6 (20)	52.6 (40)	48.8 (60)
	Memory disorder	37.0 (10)	20.0 (4)	29.8 (14)
**Do you consider having received enough information on the disposal of medical patches?**		***n*** **=** **96**	***n*** **=** **127**	***n*** **=** **223**
		**77.1 (74)**	**85.0 (108)**	**81.7 (182)**
**Gender**				
Female		73.9 (51)	83.5 (76)	79.4 (127)
Male		83.3 (20)	88.6 (31)	86.4 (51)
**Age (years)**				
−45		44.4 (4)	78.6 (11)	65.2 (15)
46–65		84.1 (37)	85.9 (55)	85.2 (92)
66–		76.7 (33)	85.7 (42)	81.5 (75)
**Type of the medical patch**		*p* = 0.035	*p* < 0.001	*p* < 0.001
Opioid		85.7 (54)	89.6 (69)	87.9 (123)
Hormonal		63.6 (14)	85.0 (34)	77.4 (48)
Memory disorder		60.0 (6)	16.7 (1)	43.8 (7)

Of the respondents, 54.9% (96/175) of respondents in 2022 and 52.3% (127/243) answered this question. Of these respondents, 77.1% in 2022, 85.0% in 2023reported that they had received sufficient information about the disposal of medical patches. There were no statistically significant differences between background variables or the years 2022 and 2023.

### 3.3 Information sources

**Fig. 1:**
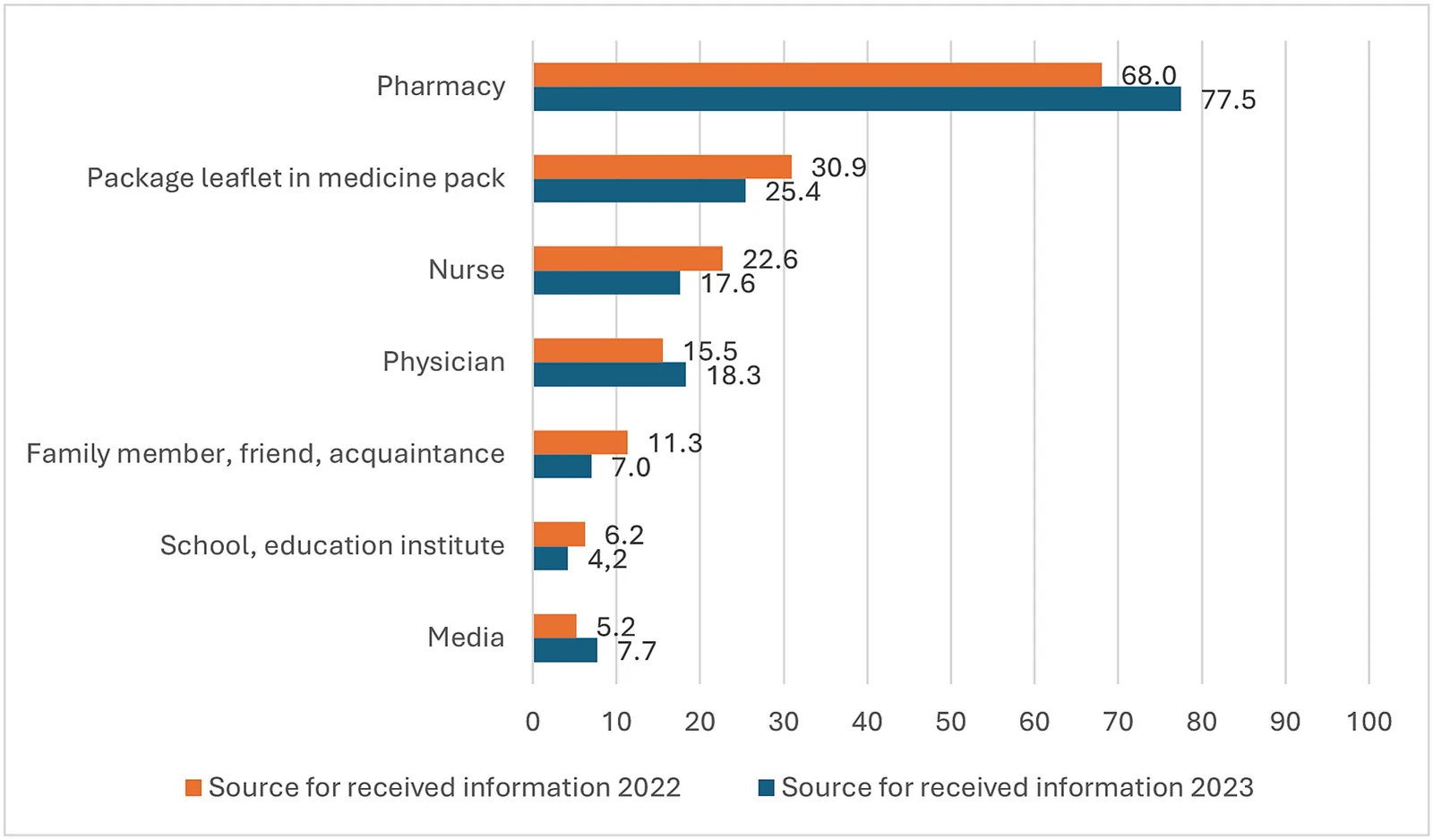
Information sources for received information about disposal of medical patches among the respondents who answered that they have had information about disposal of medical patches in general (*N* = 239). A respondent may have chosen multiple options.

Pharmacy (73.6% in total) was the most common information source for the disposal of medical patches among the respondents (Fig. 1). Media (6.7% in total) and schools or educational institutes (5.0% in total) were the least common sources of information. Information sources differed significantly by both age and patch type. Younger respondents (−45 years) were more likely than older age groups to receive disposal information from package leaflets (51.9% vs. 31.9% vs. 15.6%, p < 0.001), nurses (40.7% vs. 14.7% vs. 19.8%, *p* = 0.006), families (25.9% vs. 6.0% vs. 7.3%, *p* = 0.002), and schools (11.1% vs. 6.9% vs. 1.0%, *p* = 0.027). Opioid patch users more frequently received information from pharmacies (77.6% vs. 68.2% vs. 62.5%, *p* < 0.001) and nurses (24.3% vs. 10.6% vs. 18.8%, *p* = 0.013) compared to hormonal or memory disorder patch users (Table B.1). There were no statistically significant differences between the years 2022 and 2023.

**Fig. 2:**
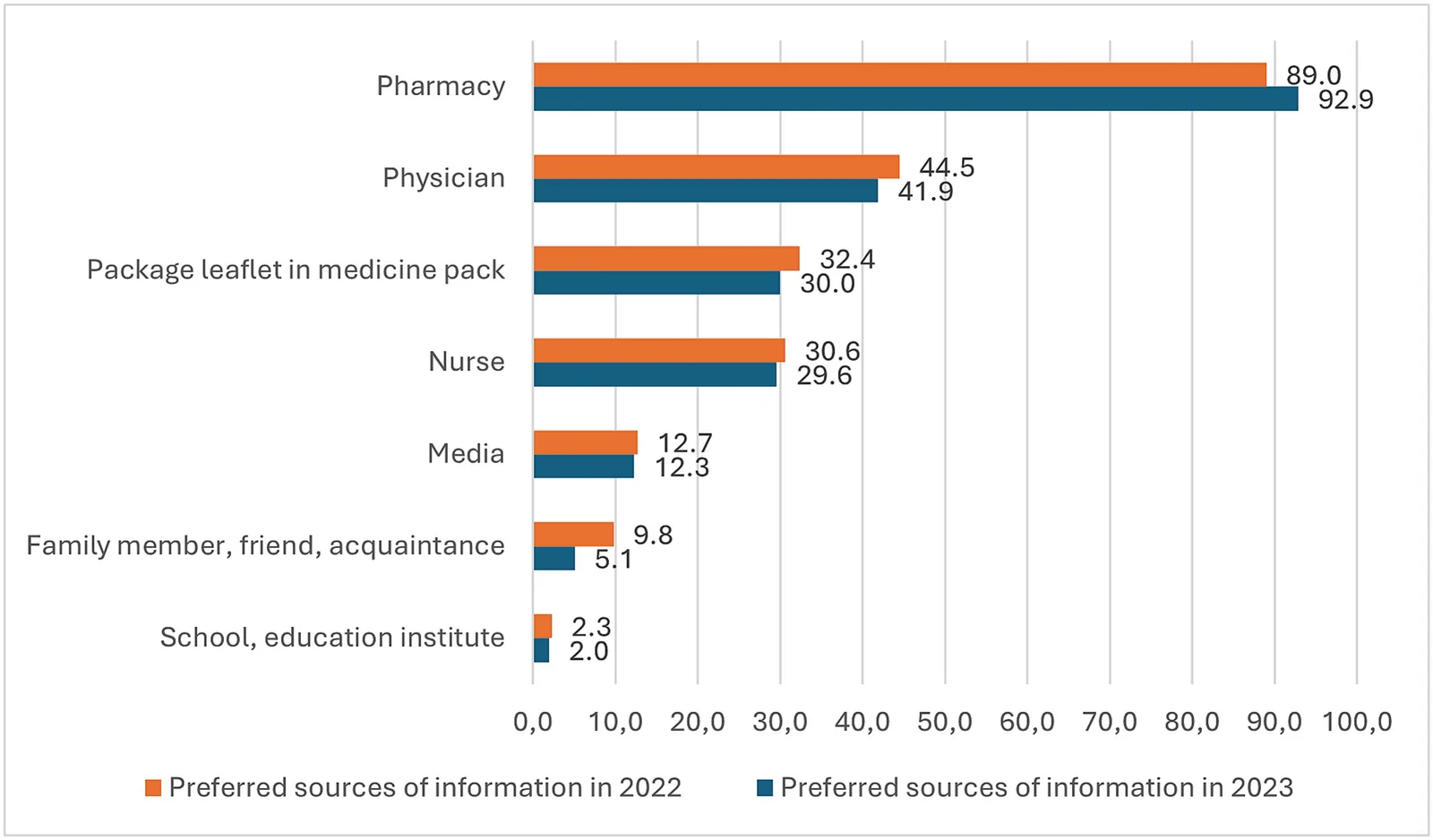
Preferred sources of information about disposal of medical patches among the respondents (*N* = 426). A respondent may have chosen multiple options.

Of the respondents, 91.3% preferred pharmacies, 43.0% physicians, 31.0% package leaflets, and 30.0% nurses as their primary information source for the disposal of medical patches in total (Fig. 2).

Pharmacy was equally preferred across all age groups (range 83.7–93.4%), but preferences for other information sources differed significantly. Respondents in the youngest age group (−45 years) were more likely than older respondents to prefer information from physicians (69.8% vs. 43.9% vs. 35.7%, *p* < 0.001), package leaflets (58.1% vs. 37.4% vs. 17.8%, *p* < 0.001), nurses (58.1% vs. 28.3% vs. 25.4%, *p* < 0.001), media (23.3% vs. 13.6% vs. 8.6%, *p* = 0.038), family members (20.9% vs. 6.1% vs. 4.8%, *p* < 0.001), and schools (9.3% vs. 2.0% vs. 0.5%, *p* = 0.002) (Table B.2). In addition, hormonal patch users more frequently preferred information from pharmacies (95.1% vs. 91.3% vs. 83.3%, *p* = 0.022) and package leaflets (43.1% vs. 28.1% vs. 12.5%, *p* < 0.001) compared to opioid and memory disorder patch users. There were no statistically significant differences between the years 2022 and 2023.

## 4 Discussion

The findings of this study highlight the challenges associated with the proper disposal of medical patches and emphasise the critical role of pharmacies in raising public awareness about environmentally friendly disposal practices of medical patches. While disposing of pharmaceuticals via mixed waste and sewers are globally common practice,^22^ in Finland, people commonly follow the correct disposal methods for pharmaceuticals by returning them to a pharmacy.^10^ However, based on our study, this is not the case for medical patches: less than half of the respondents stated to return their used or unneeded medical patches to a pharmacy for disposal. This emphasises the persistence of the knowledge–action gap regarding the correct disposal of medical patches.

The disposal practices observed in this study reveal challenges in appropriate medical patch disposal. While respondents demonstrated higher return rates for unused patches compared to used ones, and a slight improvement was noted between 2022 and 2023, the overall proportion returning patches to pharmacies was low. Interestingly, opioid patches were most frequently returned to pharmacies, probably reflecting heightened public awareness of the health and safety risks associated with these products. This aligns with prior research suggesting that risk perceptions influence people's medicine disposal behaviours,^23^ though studies specifically focusing on disposal practices of medical patches have not been conducted. One of the reasons behind the tendency of returning opioid patches to pharmacies may be the broad news coverage and general knowledge regarding the risks associated with opioid patches.^24^, ^25^ Given the low return rates of medical patches to pharmacies, they currently seem to represent a group of pharmaceutical products for which the importance of proper disposal should be emphasised to minimise the numerous risks associated with improper disposal. Such risks include accidental exposure to the active ingredients in medicinal patches, their misuse, release of pharmaceutical residues into the environment, and their diverse and often unpredictable impacts on the environment.

Approximately half of the respondents recalled having received information on the disposal of medical patches, with slightly higher rates in 2023 compared to 2022, though the differences were not statistically significant. Among those who had received information, the majority (82%) considered it sufficient, though this translates to only 43.5% of all respondents feeling adequately informed about proper disposal practices. Notably, individuals using memory disorder patches were less likely to report having received such information, and, even when they had, were significantly less likely to consider it sufficient. This may reflect challenges unique to this group, particularly if the respondents themselves were affected by cognitive and memory impairments, or if caregivers lack access to adequate disposal guidance.

Pharmacies were the predominant source of information about proper disposal of medical patches in both 2022 (68%) and 2023 (78%), followed by package leaflets and healthcare professionals such as nurses and physicians. This pattern of pharmacies and package leaflets being the most frequent information sources is in line with previous studies on patients' information sources related to medicines in general^26^ and information related to the environmental impacts of pharmaceuticals.^27^ In our study, younger respondents were more likely to obtain information from package leaflets, educational institutions, or social networks, indicating generational differences in information-seeking behaviours compared to older respondents. Younger people may be more proactive and independent in accessing medication-related information, which should also be considered when providing information on different platforms. Given the widespread use of package leaflets regarding the use and disposal of medicines, regulatory changes to enhance their content could be a highly impactful measure to improve disposal practices. Electronic package leaflets could include access to more in depth online information resources and might improve accessibility to such information, thereby supporting environmentally friendly disposal practices among many other sustainability advantages regarding the pharmaceutical sector.^28^

According to our study, pharmacies are the preferred source of information for medical patch disposal instructions. In 2022, 89% of respondents stated pharmacies as their preferred information source, the share being 93% in 2023. Given the high frequency of patient-pharmacy interactions during medication dispensing, pharmacies are uniquely positioned to effectively disseminate information on proper disposal practices. However, results from our study indicate that a half-year-long pharmacy-based information campaign might not be enough to improve disposal behaviours or awareness among the public, highlighting the need for sustained and multifaceted communication efforts. Future campaigns should use diverse communication channels to reach different demographic groups effectively. For instance, younger users accustomed to digital platforms and social media could be targeted through tailored online-based communication, while the older population might benefit from more traditional communication methods. In the context of information on pharmaceutical waste, particular attention should be paid to medical patches as pharmaceuticals since they may have significant environmental impacts. Therefore, healthcare professionals must have sufficient knowledge, ability, and motivation to raise the question of environmentally sound use of pharmaceuticals, including medical patches, when encountering patients.

### 4.1 Strengths and limitations

This study addresses a critical research gap, as there is a lack of prior research into the disposal practices of medical patches. While our findings are most applicable to countries where pharmaceutical waste collection is managed through pharmacies, such as in many EU member states, the study's insights have broader implications for promoting environmentally friendly pharmaceutical practices. Our study survey targeted pharmacy customers receiving medical patches, which ensures the relevance of the study sample to the study objectives. Moreover, practical factors, such as the survey environment (pharmacies), may have influenced responses, potentially framing respondents' focus on pharmaceutical issues. Because data were collected immediately after a counselling interaction, there is a risk of social desirability bias, given that proper disposal is a socially desirable, normative behaviour in a pharmacy setting. This may have led some respondents to over-report correct disposal practices. Despite this, the relatively low rates of proper disposal reported by the respondents suggest that they provided honest answers rather than ones they could consider correct or desired. The sample size, consisting of 430 respondents across two years, limits the statistical power and necessitates a cautious interpretation of the results. It is also worth noticing that our research design does not guarantee that a certain individual respondent had been exposed to the information campaign or had remained involved in the survey, which prevents us from concluding the real-world impact of an information campaign on disposal practices. In addition, survey respondents were chosen among the pharmacy customers receiving prescription medical patches, thereby excluding those who use medical patches without a prescription, such as nicotine patches. Nicotine replacement products, including nicotine patches, are the most-sold pharmaceuticals in Finland, and unlike other medicines, they are also sold outside pharmacies.^29^

### 4.2 Future research

In the future, study designs that could measure the impact of information campaigns on the correct disposal of medical patches are warranted. This topic would benefit from using validated, more diverse survey instruments to capture knowledge, attitudes, and practices specific to medicine disposal and handling of medical patches. In addition, mixed-methods approaches could combine quantitative tracking of behaviour with interviews to identify and explain barriers to correct disposal practices to inform the needs for the communication measures.

## 5 Conclusion

There is a need for improvement in the proper disposal practices of medical patches, even though people are generally aware of the proper disposal of medicines. Enhanced communication on the environmental impact and the proper disposal of medical patches is essential to mitigate the environmental risks of pharmaceuticals. Although the pharmacy-based campaign showed some promise, it underscores the need for multifaceted long-term efforts to address knowledge gaps and barriers to action by the users of medicines. Tailoring communication strategies to diverse demographic groups is a critical step toward more sustainable pharmaceutical practices. To achieve this, it is essential that all healthcare professionals and stakeholders focus on including environmental aspects in patient care and disseminate this information actively through different channels.

## CRediT authorship contribution statement

**Lasse Alajärvi:** Writing – original draft. **Outi Lapatto-Reiniluoto:** Writing – review & editing, Methodology, Conceptualization. **Henna Kyllönen:** Writing – review & editing, Methodology, Conceptualization. **Piia Siitonen:** Writing – review & editing, Writing – original draft, Methodology, Formal analysis, Data curation, Conceptualization.

## Funding sources

This research did not receive any specific grant from funding agencies in the public, commercial, or not-for-profit sectors.

## Declaration of competing interest

The authors declare that they have no known competing financial interests or personal relationships that could have appeared to influence the work reported in this paper.

## Figures and Tables

**Table 1:** Information sources for received information about disposal of medical patches among the respondents who answered that they have had information about disposal of medical patches in general (*n* = 239) according to background variables (only statistically significant differences are shown). A respondent may have chosen multiple options.

		Gender (*n* = 233)		Age (*n* = 239)			Type of medical patch (*n* = 234)		
	Total % (*n*)	Male	Female	−45	46–65	66-	Opioid	Hormonal	Memory disorder
**Pharmacy**	**73.6 (176)**	72.6 (45)	73.7(126)	63.0 (17)	75.9 (88)	74.0 (71)	77.6 (118)	68.2 (45)	62.5 (10)
*p*-value							*<0.001*		
**Package leaflet**	**27.6 (66)**	21.0 (13)	29.8 (51)	51.9 (14)	31.9 (37)	15.6 (15)	27.0 (41)	31.8 (21)	18.8 (3)
*p*-value				*<0.001*					
**Nurse**	**19.7 (47)**	22.6 (14)	18.1 (31)	40.7 (11)	14.7 (17)	19.8 (19)	24.3 (37)	10.6 (7)	18.8 (3)
*p*-value				*0.006*			*0.013*		
**Physician**	**17.2 (41)**	21.0 (13)	16.4 (28)	29.6 (8)	14.7 (17)	16.7 (16)	17.1 (26)	18.2 (12)	18.8(3)
p-value									
**Family member, friend, acquitance**	**8.8 (21)**	8.1 (5)	9.4 (16)	25.9 (7)	6.0 (7)	7.3 (7)	8.6 (13)	6.1 (4)	12.5 (2)
*p*-value				*0.002*					
**Media**	**6.7 (16)**	3.2 (2)	8.2 (14)	7.4 (2)	8.6 (10)	4.2 (4)	5.3 (8)	12.1 (8)	0 (0)
*p*-value									
**School, educational institute**	**5.0 (12)**	0 (0)	7.0 (12)	11.1 (3)	6.9 (8)	1.0 (1)	3.9 (6)	6.1 (4)	12.5 (2)
*p*-value		*0.042*		*0.027*					

**Table 2:** Preferred information sources about the disposal of medical patches among the respondents (*N* = 426) according to background variables (only statistically significant differences are shown). A respondent may have chosen multiple options.

		Gender		Age			Type of medical patch		
	Total % (*n*)	Male	Female	−45	46–65	66-	Opioid	Hormonal	Memory disorder
**Pharmacy**	91.3 (389)	89.8 (97)	91.7 (286)	83.7 (36)	93.4 (185)	90.8 (168)	91.3 (221)	95.1 (117)	83.3 (40)
*p*-value							*0.022*		
**Physician**	43.0 (183)	49.1 (53)	40.4 (126)	69.8 (30)	43.9 (87)	35.7 (66)	45.0 (109)	42.3 (52)	31.3 (15)
*p*-value				*<0.001*					
**Package leaflet**	31.0 (132)	25.9 (28)	32.1 (100)	58.1 (25)	37.4 (74)	17.8 (33)	28.1 (68)	43.1 (53)	12.5 (6)
*p*-value				*<0.001*			*<0.001*		
**Nurse**	30.0 (128)	33.3 (36)	28.8 (90)	58.1 (25)	28.3 (56)	25.4 (47)	33.5 (81)	23.6 (29)	27.1 (13)
*p*-value				*<0.001*					
**Media**	12.4 (53)	8.3 (9)	13.8 (43)	23.3 (10)	13.6 (27)	8.6 (16)	10.7 (26)	17.1 (21)	6.3 (3)
*p*-value				*0.08*			*0.080*		
**Family member, friend, acquitance**	7.0 (30)	12.0 (13)	5.1 (16)	20.9 (9)	6.1 (12)	4.8 (9)	7.4 (18)	4.1 (5)	6.3 (3)
*p*-value		*0.018*		*<0.001*					
**School, educational institute**	2.1 (9)	1.9 (2)	1.9 (6)	9.3 (4)	2.0 (4)	0.5 (1)	2.5 (6)	2.4 (3)	0 (0)
*p*-value				*0.002*					

## Data Availability

The data presented in this study are available on request from the corresponding author.

## Appendix A Appendix

### A.1 Assignment instructions for pharmacy students and the customer questionnaire

The objective of this assignment is to survey customers' practices and experiences regarding the disposal of medical patches and related counselling.

Aim to conduct at least two customer counselling situations. The customer may be a person collecting medical patches by prescription for themselves or a family member. Ask the customer if they would like to respond to a brief survey about customers' practices and experiences regarding the disposal of medical patches and related counselling. Additionally, explain that the survey is part of an internship assignment and that responses are collected anonymously.

Provide the customer with a paper questionnaire form that they complete anonymously after the dispensing situation. When the customer returns the completed form to you, inform them about the proper disposal of medical patches and explain why their appropriate disposal is important. Please note that you should advise the customer neutrally and without making them feel guilty, in accordance with motivational conversation principles, even if they have not previously disposed of medical patches appropriately.

Write the active pharmaceutical ingredient of the medical patch on the form. Transfer the customers' responses to electronic format by completing the electronical survey in eLearn Moodle separately for each customer. Dispose of the customer's completed form securely, even though the form does not contain the customer's personal information.

### A.2 Disposal practices of medical patches and information sources

1. How do you usually dispose of used medical patches?

1. I return them to the pharmacy
2. I put them in household waste
3. I flus them down the drain
4. I give them to others for use
5. Other, please specify

2. How would you dispose of unused or expired medical patches?

1. I return them to the pharmacy
2. I put them in household waste
3. I flus them down the drain
4. I give them to others for use
5. Other, please specify

3. Have you been previously informed about the disposal of medical patches?

1. No (Go to the questions 6.)
2. Yes
3. I don't know

4. From where/whom have you received information about disposal of medical parches? You can select several options.

1. Doctor
2. Nurse
3. Pharmacy
4. Family members friend, acquaintance
5. School or educational institution
6. Package leaflet in medicine pack
7. Internet site
8. Social media
9. Media (e.g. television, radio, newspapers)
10. Other sources, please specify

5. Do you consider having received enough information on the disposal of medical patches?

1. Yes
2. No
3. I don't know

6. From which sources would you rather receive information about disposal of medical parches? You can select several options.

1. Doctor
2. Nurse
3. Pharmacy
4. Family members friend, acquaintance
5. School or educational institution
6. Package leaflet in medicine pack
7. Internet site
8. Social media
9. Media (e.g. television, radio, newspapers)
10. Other sources, please specify

**Background information of the respondent**

Gender

1. Female
2. Male
3. Other
4. I don't want to answer

Age______years

**To be completed be the pharmacy student:**

Active pharmaceutical ingredient of the medical patch:____________________
